# Anatomical variation of the Psoas Valley: a scoping review

**DOI:** 10.1186/s12891-020-03241-1

**Published:** 2020-04-10

**Authors:** Yuichi Kuroda, Ankit Rai, Masayoshi Saito, Vikas Khanduja

**Affiliations:** 1 Young Adult Hip Service, Department of Trauma and Orthopaedic Surgery, Addenbrooke’s-Cambridge University Hospitals NHS Foundation Trust, Box 37, Hills Road, Cambridge, CB2 0QQ UK; 2 University of Cambridge, Cambridge, UK

**Keywords:** Psoas-U, Anterior depression, Iliopsoas impingement, Iliopsoas notch

## Abstract

**Background:**

This scoping review aimed to investigate the literature on the anatomy of the psoas valley, an anterior depression on the acetabular rim, and propose a unified definition of the anatomical structure, describe its dimensions, anatomical variations and clinical implications.

**Methods:**

A systematic computer search of EMBASE, PubMed and Cochrane for literature related to the psoas valley was undertaken using Preferred Reporting Items for Systematic Reviews and Meta-analyses (PRISMA) guidelines. Clinical outcome studies, prospective/retrospective case series, case reports and review articles that described the psoas valley and its synonyms were included. Studies on animals as well as book chapters were excluded.

**Results:**

Of the 313 articles, the filtered literature search identified 14 papers describing the psoas valley and its synonyms such as iliopsoas notch, a notch between anterior inferior iliac spine and the iliopubic eminence, Psoas-U and anterior wall depression. Most of these were cross-sectional studies that mainly analyzed normal skeletal hips. In terms of anatomical variation, 4 different configurations of the anterior acetabular rim have been identified and it was found that the curved type was the most frequent while the straight type may be nonexistent. Additionally, the psoas valley tended to be deeper in males as compared with females. Several papers established the psoas valley, or Psoas-U in a consistent location at approximately 3 o’clock on the acetabular rim which may have implications with labral pathology.

**Conclusion:**

This review highlights the importance of the anatomy of the psoas valley which is a consistent bony landmark. The anatomy and the anatomical variations of the psoas valley need to be well-appreciated by surgeons involved in the management of young adults with hip pathology and also joint replacement surgeons to ensure appropriate seating of the acetabular component.

## Background

The depression of the anterior acetabular rim, the so-called ‘psoas valley’ has assumed significant clinical importance in recent times, but is poorly understood and reported from an anatomical point of view [1, 2]. The psoas valley acts as a groove anteriorly for the passage of the iliopsoas muscle as it tracts over the acetabular rim and its location coincides with a site commonly associated with acetabular labral pathologies [3]. Philippon et al. recognized the anterior labral sulcus as a concave impression on the anterior rim of the acetabulum, and also note the location of the iliopsoas tendon anteriorly [4]. Consistent with these factors, it is likely that iliopsoas impingement, rather than femoroacetabular impingement (FAI), is the key player in the development of focal labral tears in this location [5, 6].

In performing a total hip replacement (THR), the geometric discrepancy between hemispherical implants and the native acetabular morphology including the psoas valley often results in a partial prosthetic overlap of the acetabular rim [2]. This overlap can manifest in a pathology known as iliopsoas impingement through chronic friction between the iliopsoas tendon and the rim of the implant [7]. Therefore, a description of the natural deviations from a hemispherical rim is an area of interest for hip replacement surgeons and implant manufacturers [2, 7].

However, the complexity of acetabular architecture and biomechanics may contribute to the limited progress of anatomical studies in this area [3, 8], hence most health-care professionals do not have a comprehensive grasp on the psoas valley at present. Thus, this scoping review was conducted to systematically investigate the literature on the anatomy of the psoas valley and propose a unified definition of the anatomical structure, describe its dimensions, anatomical variations and clinical implications to eventually help manage our patients better.

## Methods

We conducted a scoping review using the method outlined by Arksey and O’Malley [9].

This review style has been designed to be broad enough to include any types of existing scientific literature, thus allowing the most complete mapping on the desired subjects and allowing to better answer the research questions in this study.

### Research question

**Fig. 1:**
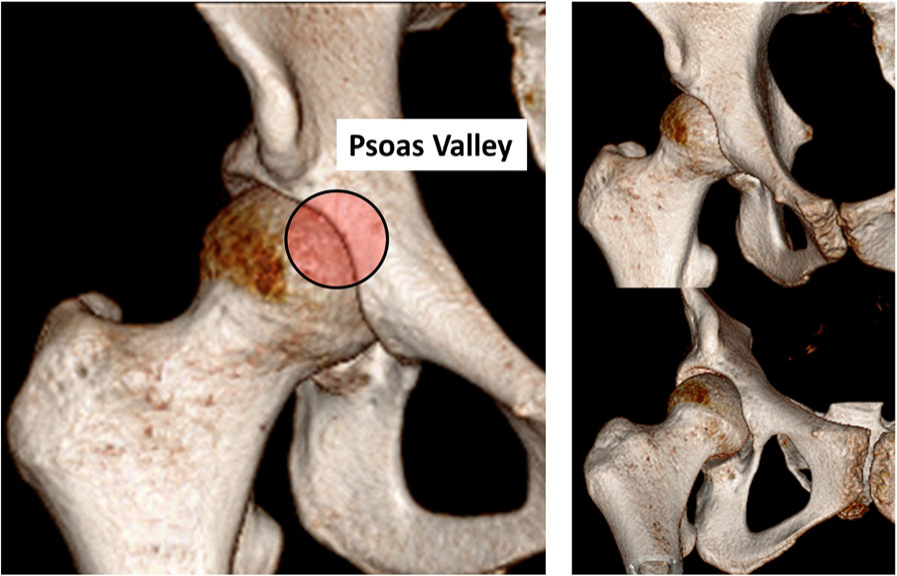
Psoas valley in 3D-CT scan

**Fig. 2:**
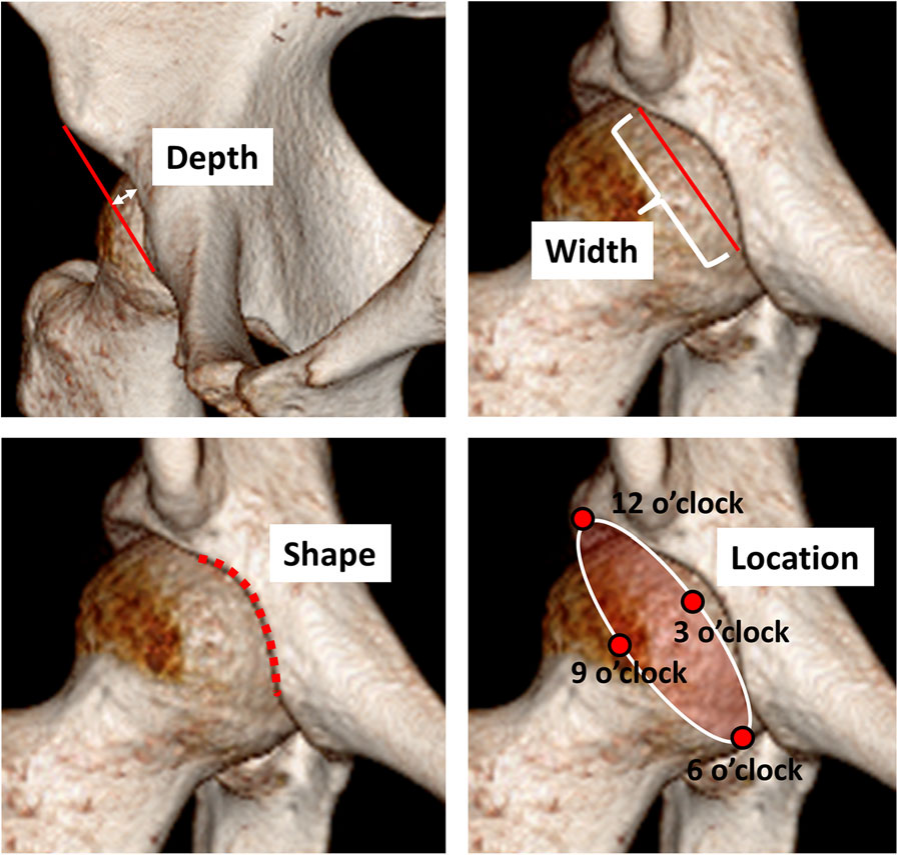
Dimensions of the psoas valley in 3D-CT scan

The scoping review aimed to answer the following primary question: “What is known about the anatomical variations of the psoas valley? **(**Fig. 1**)**” The secondary questions were set as follows;

1. How is the psoas valley defined in the literature?
2. What are the dimensions of the psoas valley i.e. depth, width, shape and location? **(**Fig. 2**)** and finally
3. What are the factors which influence the anatomy of the psoas valley?

### Identification of studies

A systematic computer search of EMBASE, PubMed and Cochrane for literature including variations, descriptions, classification or measurements of the iliopsoas valley was conducted on the 7th of October 2019. Each database was searched from inception to October 2019 using MeSH terms and keywords relating to: anatomical (including anatomy, anatomical, morphological or skeletal), psoas valley (including psoas valley, psoas notch, psoas depression, anterior labral sulcus, notch, valley, depression or sulcus), anterior (including anterior or anterosuperior) and hip (including hip, acetabul* or pelvi*) **(**Additional file 1**)**.

### Eligibility criteria

The eligibility criteria for literature was determined before the search by the authors. Inclusion criteria specified both retrospective and prospective studies, case reports, case series and randomized controlled trials. Studies were included if they were conducted on human subjects (all ages, both sexes, symptomatic and asymptomatic), cadavers, and if they were concerned with the hip and more specifically, the depression of the anterior acetabular rim, the so-called psoas valley. Descriptions of surgical technique involving consideration of the psoas valley as well as expert opinions on the anatomy of the psoas valley were also included. Studies on animals were excluded, as well as those reported in languages other than English. Papers concentrating on joints other than the hip were excluded as well, along with those in which the inferior and/or posterior valleys of the acetabulum, rather than the psoas valley, were the key focus. Additionally, book chapters were not considered.

### Screening, eligibility and inclusion

Two independent reviewers completed the title, abstract and full-text screening assessing for study inclusion, and any discrepancies were resolved by discussion. A third party was involved if there was no mutual agreement.

### Data extraction

The definition of psoas valley (or equivalent term), sample subjects and measurements of the psoas valley were extracted from each study. Further information relating to the anatomy of the psoas valley was also acquired from the studies, and a descriptive analysis of the information gathered was performed. The above-described review process and the exclusion criteria were performed in concordance with current Preferred Reporting Items for Systematic Reviews and Meta-analyses (PRISMA) guidelines [10] for scoping reviews.

### Statistical analysis

The extracted evidence was collected and analyzed with Microsoft Excel 2013 (Microsoft Corporation, Redmond, Washington). Statistical analyses focused on descriptive statistics.

## Results

A total of 313 articles were identified in the original search, and two further articles were gleaned from other sources. After the removal of duplicates between databases, a total of 245 records were identified for title screening. After the exclusion of 157 papers based on title, 88 records remained for abstract screening, of which 36 full texts were deemed relevant based on the inclusion and exclusion criteria.

**Fig. 3:**
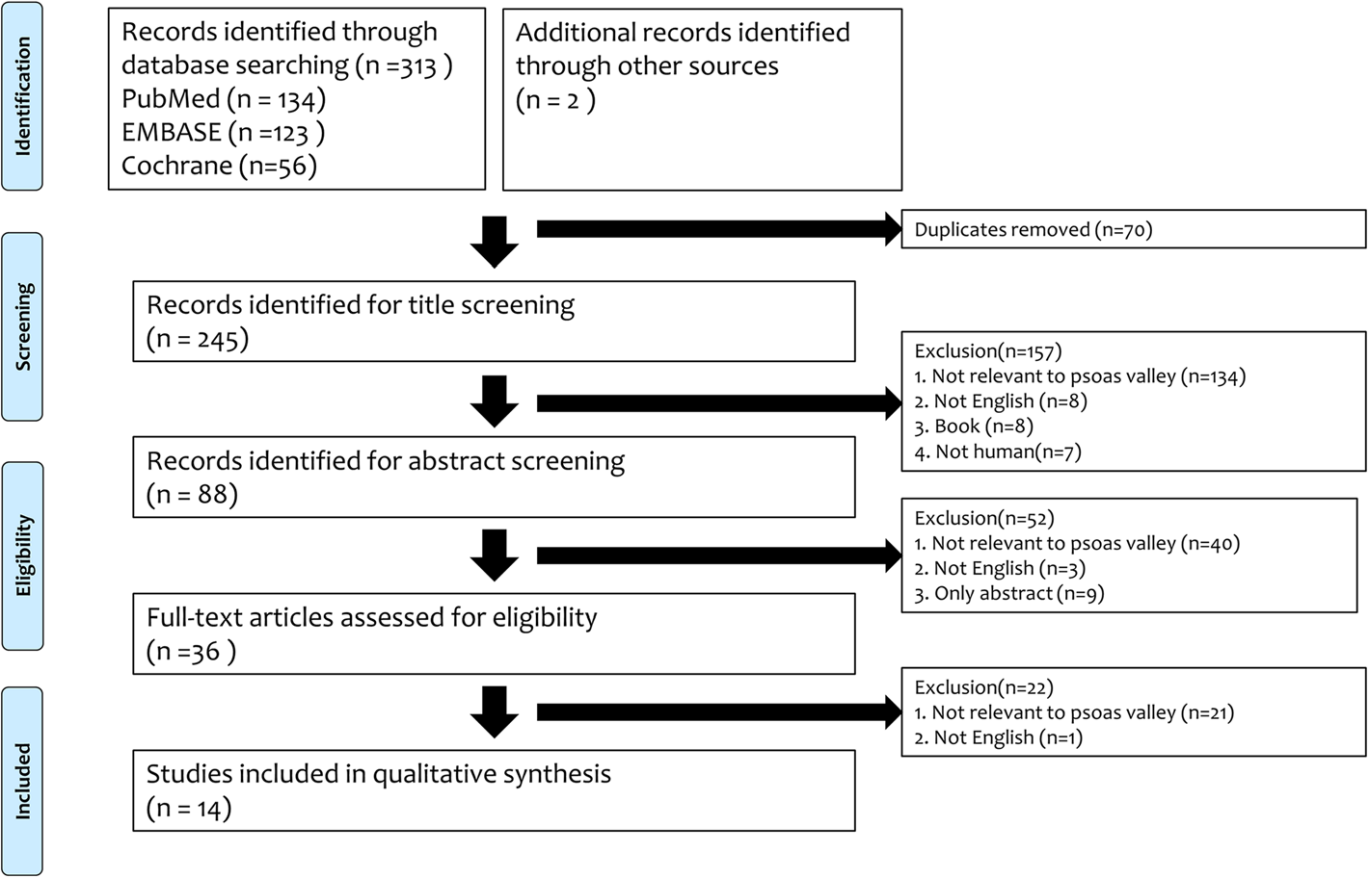
PRISMA (Preferred Reporting Items for Systematic Reviews and Meta-analyses) flow diagram of study inclusion

Assessment of these full-text articles yielded 14 final articles for qualitative analysis **(**Fig. 3**)** [1–5, 11–19]. Publication dates ranged from 1992 to 2016.

Only one of these articles was longitudinal [5] whist 12 were cross-sectional. The remaining article was a description of a surgical technique [19]. Seven of the articles reported manual measurements of the pelvis [3, 11–14, 16, 18] whereas 5 studies took measurements with radiographic images, a navigation system or imaging software [1, 2, 4, 15, 17] **(**Table 1**)**.

**Table 1:** Details of 14 studies included in the scoping review

Publication year	First author	Subjects	Definition	Depth	Width	Shape	Location	Index of widening	Measurement method
2016	Spiker [19]	Introduction of surgical technique	Iliopsoas notch						
2016	Lee [15]	10 fresh-frozen cadaveric specimens	Psoas-U	26.9 ± 2.6 mm proximal to the tear drop (AP view) 22.9 ± 4.8 mm anterior to the center of the acetabulum (FP view)					Radiography
2014	Philippon [4]	14 fresh-frozen cadaveric specimens	Psoas-U				3:30′ in o’clockface position superior-most point: 23.4 ± 2.9 mm from AIIS center: 29.4 ± 3.4 mm from AIIS		Coordinate-measuring device
2014	Kopydlowski [3]	240 human skeletons	Psoas valley	4.64 ± 1.62 mm	26.94 ± 5.03 mm		3.92 ± 0.42 o’clock anteriorly and 2.12 ± 0.77 o’clock posteriorly in clockface position		Manual
2014	Devi [11]	100 human skeletons				Curved:60% Angular: 27% Irregular: 9% Straight: 4%			Manual
2013	Osmani [17]	65 CT scans of non-diseased hips	Psoas valley						3D imaging software
2011	Sachdeva [18]	50 cadaveric specimens	Notch between AIIS and IE	1.26 ± 0.3 cm in male 1.02 ± 0.18 cm in female				30.06 ± 6.72 in male 26.9 ± 6.06 in female	Manual
2011	Domb [5]	36 patients who underwent hip arthroscopy	Iliopsoas notch						
2009	Kohnlein [14]	33 human skeletons	Anterior wall depression	81 ± 5° of geographical reconstruction method 9° below the level of a hemisphere		Irregular (wave-like rim profile) Straight: 0%	03: 20 ± 20 min in clockface position		Manual
2008	Vandenbussche [2]	200 CT scans of non-diseased hips	Psoas valley	4.9 ± 1.6 mm		Curved:79% Angular: 11% Irregular: 10% Straight: 0%			Image processing software
2007	Vandenbussche [1]	34 fresh cadaveric specimens	Psoas valley	3.8 ± 2.0 mm	71 ± 18°	Curved:58% Angular: 22% Irregular: 17% Straight: 3%			Surgical navigation system
2005	Govsa [13]	226 human skeletons				Curved:60.5% Angular: 25.5% Irregular: 9.5% Straight: 4.5%			Manual
2001	Maruyama [16]	100 human skeletons				Curved:60.5% Angular: 25.5% Irregular: 9.5% Straight: 4.5%			Manual
1992	Pellico [12]	42 human skeletons	Notch between AIIS and IE	8.13 ± 1.73 mm				21.35 ± 5.12	Manual

*AP view* anteroposterior view, *FP view* False-Profile view, *AIIS* anterior inferior iliac spine, *IE* iliopubic eminence

### Anatomy of the Psoas Valley

#### Subjects in this review

**Fig. 4:**
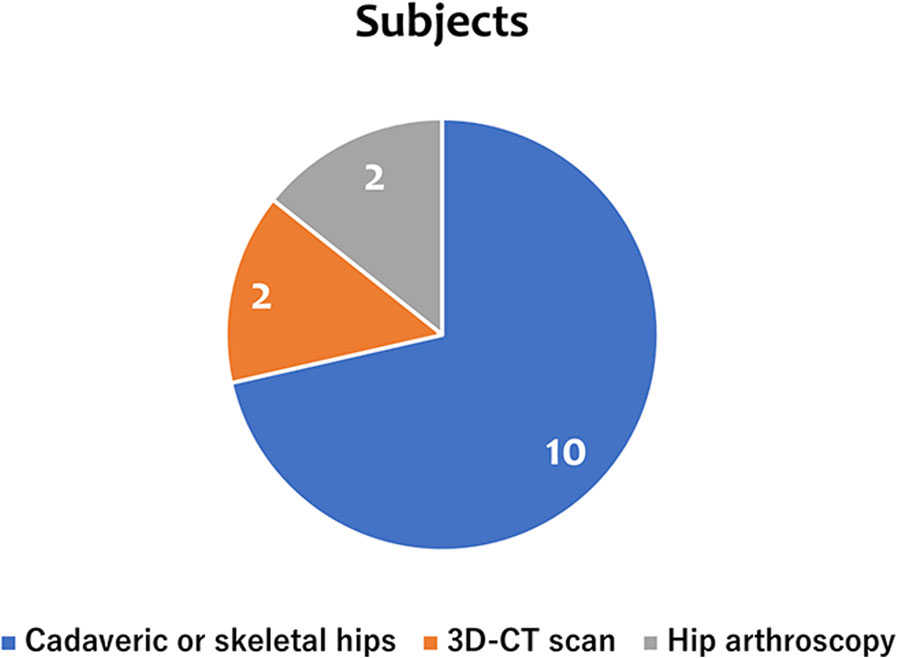
Subjects in this review

Ten articles in this review utilized cadaveric or skeletal hips from both male and female specimens [1, 3, 4, 11–16, 18]. Osmani et al. evaluated 3D-CT scans of live patients taken for colonography to make measurements of the acetabular version [17]. Similarly, Vandenbussche et al. used CT scans of live hips to quantify the psoas valley [2]. Spiker et al. introduced the technique of arthroscopic psoas management related to the iliopsoas notch [19]. Finally, Domb et al. conducted a study wherein all patients underwent hip arthroscopic surgeries due to a labral injury at the 3 o’clock position [5] **(**Table 1**) (**Fig. 4**)**.

#### Definition

The 14 papers extracted put forward differing anatomical definitions of the anterior acetabular variable we have termed the ‘psoas valley’. A categorization of these definitions was listed as below;

- The notch between the anterior inferior iliac spine and the iliopubic eminence (notch between AIIS and IE)

Two studies found a notch between the anterior inferior iliac spine (AIIS) and the iliopubic eminence (IE) [12, 18]. Pellico et al. stated that the iliopsoas muscle passes anterior to this notch [12].

b. **Psoas-U (Anterior labral sulcus)**

The Psoas-U is another term that has been used in literature to describe the psoas valley. A more descriptive term used by Philippon et al. is the superior margin of the anterior labral sulcus, which they ascribe to the 3 o’clock position, relative to the center of the acetabulum as the center of the clock face. The Psoas-U is expressed as a concave impression of the anterior rim of the acetabulum, and relates anteriorly to the iliopsoas tendon [4]. Both Philippon et al. and Lee et al. vouch for the consistency of the Psoas-U 3 o’clock location, and in fact both use it as the key reference point in creating the clock face depiction of the acetabulum [4, 15]. Thus, like the previous ‘notch’ description, the Psoas-U has bony specifications and is related to the iliopsoas muscle, but it adds a cartilaginous bearing and has a more specific position in relation to the acetabulum.

c. **Anterior wall depression**

Only 1 study described an anterior wall depression. Kohnlein et al. detected three constant prominences and two depressions on the acetabular bony rim, and defined one of the depressions as the anterior wall depression [14].

d. **Psoas valley**

Four studies defined the ‘psoas valley’ [1–3, 17]. Building on Maruyama et al’s work in distinguishing qualitative configurations of the anterior acetabular rim [16], Vandenbussche’s group aimed to quantify variations in acetabular rim morphology in cadavers, with particular focus on the psoas valley [1]. In the studies included in our review, they introduced the term ‘psoas valley’.

e. **Iliopsoas notch**

Two papers found in the search do not extensively describe the psoas valley, and simply refer to it as the iliopsoas notch at the 3 o’clock position [5, 19].

#### Measurement

- **Depth**

Six studies described the depth of the psoas valley and its anatomically equivalent notch [1–3, 12, 14, 18]. Pellico et al. and Sachdeva et al. defined the notch between the AIIS and IE, and measured it in a similar manner using calipers [12, 18]. The results were reported as 0.82 ± 0.16 cm and 1.26 ± 0.3 cm in males and 0.80 ± 0.195 cm and 1.02 ± 0.18 cm in females, respectively. Sachdeva et al. proposed that the different results in the two studies may be attributed to the different sets of populations, i.e. Spanish [12] and North Indians [18].

Kohnlein et al. expressed the values of the anterior depression in degrees; as a function of a geographical reconstruction of the acetabulum [14]. The pole of the acetabular hemisphere represented 0°, and the depth of the cup is tantamount to the latitude. The circle at 90° marks the equatorial level of the hemisphere. In the above measurement, the depth of the anterior depression was 81 ± 5 °, in other words, 9° below the level of a hemisphere.

Kopydlowski et al. used the distance between a ruler, placed lateral to the 2 bony peaks that border the psoas valley, and the deepest point of the psoas valley (determined using a caliper) as a measurement of depth which came out to be 4.64 ± 1.62 mm [3]. Vandenbussche et al. established a pelvic coordinate system with the origin at the midpoint between the anterior superior iliac spines to analyze the position of the psoas valley along the acetabular rim [1, 2]. On each acetabulum, the articular surface and rim were digitized with a certain number of points. Using this coordinate system and digitalized data, a two-dimensional plot of each individual acetabulum was produced with each point of inflexion labelled. The depth was defined as the distance between the trough of the psoas valley and the average heights of adjacent peaks [1] or between the psoas valley trough and the mean acetabular equator [2] which correspond to the above inflection points. These values were 3.8 ± 2.0 mm and 4.9 ± 2.0 mm, respectively. **(**Table 1**).**

b. **Width**

The width was quantified in 2 papers [1, 3]. Vandenbussche et al. used the aforementioned coordinate system and defined the angle between two peaks adjacent to the psoas valley as its width, which was 71.0 ± 18.0° [1]. In another study, a ruler was placed lateral to the 2 bony peaks that border the psoas valley on the acetabular rim, and the width was measured with a digital caliper, giving a value of 26.94 ± 5.03 mm [3]. Since previous studies have reported that the distance between the AIIS and IE is about 40 mm [12, 18], it follows that the psoas valley spans more than half of this **(**Table 1**)**.

c. **Shape**

**Fig. 5:**
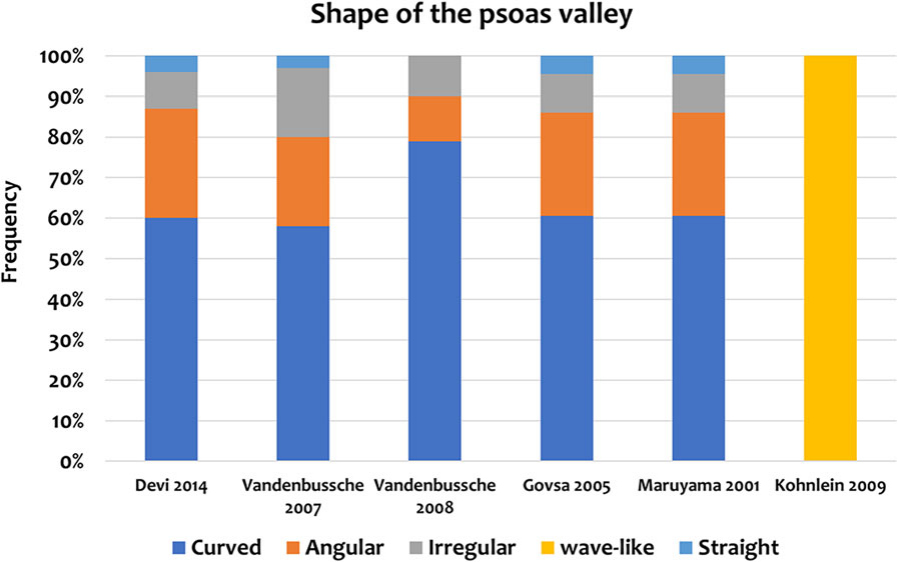
Shape of the psoas valley

Six papers described the shape of the psoas valley [1, 2, 11, 13, 14, 16]. All papers except one without a classification system, reported that the curved type was the most frequently observed configuration, seen in more than half of the subject’s acetabulae (58–79%). This was followed by angular, irregular, then straight [1, 2, 11, 13, 16]. Furthermore, two studies insinuate that a straight type may not exist [2, 14] **(**Table 1**) (**Fig. 5**)**.

d. **Location**

Four studies described the location of the psoas valley [3, 4, 14, 15]. The location of anterior wall depression is indicated in the clockwise distribution from 1:00 to 12:00 with the acetabular notch as the caudal landmark for 6:00. In this clockface representation, the location of the anterior depression was 03:20 ± 20 min [14]. With the same measurement method, Kopydlowski et al. showed that the psoas valley was located in the anterosuperior quadrant of the acetabulum, with a mean location of 3.92 ± 0.42 o’clock anteriorly and 2.12 ± 0.77 o’clock posteriorly [3]. Philippon et al. set the midpoint of the transverse acetabular ligament as the 6 o’clock position, and the Psoas-U was located at 3:30 [4]. They also measured distances from the AIIS and reported 29.4 ± 3.4 mm to the midpoint of Psoas-U and 23.4 ± 2.9 mm to the superior-most point of the Psoas-U. Lee et al., moreover, defined the Psoas-U at the 3 o’clock position and then, evaluated which position in plain radiographs corresponds to the Psoas-U in cadavers [15]. The Psoas-U was located a mean 26.9 ± 2.6 mm proximal to the teardrop in anteroposterior (AP) view and 22.9 ± 4.8 mm anterior to the vertical line drawn through the center of the acetabulum in false-profile (FP) view **(**Table 1**)**.

e. **Index of widening**

Two studies defined the index of widening as the notch depth between the AIIS and IE divided by the maximum width of this notch multiplied by 100 [12, 18]. The index values in the two studies were reported as 20.73 ± 5.12 and 30.06 ± 6.72 in males and 22.48 ± 5.08 and 26.90 ± 6.06 in females, respectively. The difference between the values of these two studies may be linked to the different ethnicities of the population as noted above **(**Table 1**)**.

h. **Related factors**

Factors related to the psoas valley and its synonyms are summarized in Table 2. The relationship with gender was the most frequently described (7 papers). Two papers reported no gender differences in depth [12, 14], while four papers showed that male valleys were deeper than female ones [1–3, 18]. Notably, no papers were reporting that the female valley was deeper. There was no significant difference reported between genders in terms of location and shape [3, 14, 16].

**Table 2:** Factors related to the psoas valley and its synonyms

First author	Definition	Gender	Age	Side	The other factors
Kopydlowski [3]	Psoas valley	Depth and width were significantly larger in male than in female. No significant difference in location.	No significant difference between younger and older in depth, width and location.		No significant difference between racial groups in depth, width and location.
Sachdeva [18]	Notch between AIIS and IE	Depth was significantly larger in male than in female. No significant difference in index of widening.		No significant difference in depth. Index of widening was significantly deeper towards the left side in male	
Kohnlein [14]	Anterior wall depression	No significant difference in depth and location.			
Vandenbussche [2]	Psoas valley	Depth was significantly larger in male than in female.	No correlation in depth		Moderate correlation between depth and acetabular diameter (R = 0.34), and between depth and anteversion (R = -0.28) were found.
Vandenbussche [1]	Psoas valley	Depth was significantly larger in male than in female. No significant difference in width.			Weak correlation between depth and acetabular diameter (R = 0.26), and between depth and anteversion (R = -0.14) were found.
Maruyama [16]		No significant difference in shape.		No significant difference in depth.	
Pellico [12]	Notch between AIIS and IE	No significant difference in depth.			

*AIIS* anterior inferior iliac spine, *IE* iliopubic eminence

Two papers evaluated the relevance of age, both reporting no correlation [2, 3]. As for differences between sides, most papers reported no significant difference, while Sachdeva et al. demonstrated that the index of widening was significantly higher on the left side [18]. However, with the same measurement method, another paper did not find a significant difference [12], hence this result may be influenced by differences among the ethnic groups of the subjects. As for other factors, Vandenbussche et al. found correlations between the depth of the psoas valley and acetabular parameters; positive with the acetabular diameter and negative with the degree of acetabular anteversion [1, 2].

## Discussion

This scoping review identified 14 studies describing the psoas valley and synonyms such as iliopsoas notch, a notch between AIIS and IE, Psoas-U and anterior wall depression. Whether these definitions are anatomically consistent is controversial, but they have similarities in that they are all related to the iliopsoas anteriorly. When positioning an acetabular component in a THR, it is important to consider the depth of the psoas valley with respect to the acetabular equator [2]. Generally, prosthetic components are medialized and elevated during a THR depending on the extent and direction of the acetabular reaming. Although medialization and elevation enable deeper seating of the cup in the acetabulum, prosthetic overlap may well persist depending on the depth of the psoas valley [2, 7]. Vandenbussche et al. reported that the depth of the psoas valley is approximately 4 mm from the acetabular equator [1, 2], which is almost the same as the degree of superomedialization in the acetabula when reaming during a THR [20]. Therefore, pre- and intra-operative assessment of the psoas valley is essential to avoid postoperative iliopsoas impingement which is becoming a major issue following arthroplasty [21–23].

Furthermore, Osmani et al. demonstrated the usefulness of the acetabular psoas valley as a measure of acetabular version angle with a 3-D CT scan [17]. Using 3-D software, the degree of acetabular version, when measured with the psoas valley and its 180° opposite counterpart rim location as landmarks, showed the same high reliability and validity as when calculated with the entire rim as a landmark. The intra-observer reliability was high (the intraclass correlation coefficient was 0.9960) and the effectiveness of using the psoas valley as a bony landmark was proven.

Several points with clinical relevance to hip arthroscopic surgery have also been reported [4, 5, 15, 19]. Iliopsoas impingement can be the source of labral pathology, which can be identified during hip arthroscopy as a characteristically focal labral lesion [24], located at the iliopsoas notch at the 3-o’ clock position [19]. Domb et al. reported that focal labral lesions at the 3 o’clock position were identified in 36 of 640 hips that had undergone hip arthroscopy in their facility [5]. In all cases, intra-operative findings revealed that the labral injury was directly adjacent to the tendinous portion of the iliopsoas muscle, and in fact in many cases, the iliopsoas was adherent or scarred to the anterior capsule.

An analysis of variability of reference points around the acetabulum in relation to the AIIS by Philippon et al. [4] revealed that the superior margin of the Psoas-U was the most consistent anatomic landmark when compared with the center of the transverse acetabular ligament and superior acetabular fossa. They propose that the Psoas-U denoting 3:00 be adopted as the new standard clock-face reference for intra-articular hip structures because of its universal presence and reliable arthroscopic visualization. Lee et al. analyzed the location of Psoas-U with anteroposterior and false-profile radiographs using cadaveric specimens [15]. This measurement was conducted using the tear drop or the center of femoral head which can be easily identified in radiographic evaluation as an index. This may be useful when performing hip arthroscopic surgery under fluoroscopic control.

The hip-spine biomechanics, morphological abnormalities like dysplasia and FAI and also abnormalities of pelvic motion have a major impact on the functioning of the Iliopsoas [25, 26].

This review is not without limitations. First, this scoping review included a wide range of study designs and methodologies, thus the level of evidence is not constant. However, does provide a detailed overview of knowledge of psoas valley and its synonyms. Second, this review consisted of several definitions such as psoas valley, psoas notch and Psoas-U, and it is unclear whether these are completely anatomically synonymous. However, there is currently no answer to this question, and it is believed that this scoping review has been able to systematically summarize the scientific knowledge that has been elucidated to date about the anterior depression of acetabular rim associated with the iliopsoas muscle.

## Conclusion

This scoping review identified that the majority of articles focused on normal skeletal hips and several analyzed anatomical parameters including depth, shape and location. It was found that the most frequent shape for the anterior acetabular rim is curved, while the straight configuration was fairly low. The psoas valley also tended to be deeper in males as compared with females. The valley was located consistently at approximately the 3 o’clock position on the acetabular rim.

Finally, this review highlights the importance of the anatomy of the psoas valley which is a consistent bony landmark. The anatomy and the anatomical variations of the psoas valley need to be well-appreciated by surgeons involved in the management of young adults with hip pathology and also joint replacement surgeons to ensure appropriate seating of the acetabular component.

## Supplementary information

**Additional file 1.** Search strategy.

## Data Availability

The datasets used and analyzed during the current study are available from the corresponding author on reasonable request.

## Abbreviations

PRISMA: Preferred reporting items for systematic reviews and meta-analyses
FAI: Femoroacetabular impingement
THR: Total hip replacement
AIIS: Anterior inferior iliac spine
IE: Iliopubic eminence
AP: Anteroposterior
FP: False-profile
