# Supplementary material for: Anatomical variation of the Psoas Valley: a scoping review
Source: BMC Musculoskelet Disord. 2020 Apr 10;21:219. doi: 10.1186/s12891-020-03241-1 (PMC7149878; doi:10.1186/s12891-020-03241-1)
Supplement: Supplementary file 1 — Additional file 1. Search strategy. [file 12891_2020_3241_MOESM1_ESM.docx]

**Additional file 1:** Search strategy

| **EMBASE: 123 Studies** | | **Cochrane: 56 Studies** | | **PubMed: 134 Studies** | |
| --- | --- | --- | --- | --- | --- |
| Strategy | Studies | Strategy | Studies | Strategy | Studies |
| 1. anatomy.mp | 242393 | 1. anatomy | 6969 | (anatomy OR anatomical OR morpholog* OR skeletal) AND (psoas valley OR psoas notch OR psoas depression OR anterior labral sulcus OR ((notch OR valley OR depression OR sulcus) AND (anterior OR anterosuperior) AND (hip OR acetabul* OR pelvi*))) | 134 |
| 2. anatomical.mp | 205261 | 2. anatomical | 5838 |  |  |
| 3. morpholog*.mp | 720956 | 3. morpholog* | 8091 |  |  |
| 4. skeletal.mp | 282513 | 4. skeletal | 15196 |  |  |
| 5. psoas valley.mp | 6 | 5. psoas valley | 1 |  |  |
| 6. psoas notch.mp | 2 | 6. psoas notch | 0 |  |  |
| 7. psoas depression.mp | 0 | 7. psoas depression | 15 |  |  |
| 8. anterior labral sulcus.mp | 2 | 8. anterior labral sulcus | 0 |  |  |
| 9. notch.mp | 36609 | 9. notch | 410 |  |  |
| 10. valley.mp | 19976 | 10. valley | 1026 |  |  |
| 11. depression.mp | 644342 | 11. depression | 70885 |  |  |
| 12. sulcus.mp | 18514 | 12. sulcus | 806 |  |  |
| 13. anterior.mp | 487921 | 13. anterior | 26472 |  |  |
| 14. anterosuperior.mp | 1439 | 14. anterosuperior | 45 |  |  |
| 15. hip.mp | 211221 | 15. hip | 21563 |  |  |
| 16. acetabul*.mp | 25963 | 16. acetabul* | 840 |  |  |
| 17. pelvi*.mp | 244105 | 17. pelvi* | 15341 |  |  |
| 18. 1 or 2 or 3 or 4 | 1352628 | 18. 1 or 2 or 3 or 4 | 34143 |  |  |
| 19. 9 or 10 or 11 or 12 | 718559 | 19. 9 or 10 or 11 or 12 | 73034 |  |  |
| 20. 13 or 14 | 488846 | 20. 13 or 14 | 26498 |  |  |
| 21. 15 or 16 or 17 | 442625 | 21. 15 or 16 or 17 | 36041 |  |  |
| 22. 19 and 20 and 21 | 341 | 22. 19 and 20 and 21 | 187 |  |  |
| 23. 5 or 6 or 7 or 8 or 22 | 342 | 23. 5 or 6 or 7 or 8 or 22 | 199 |  |  |
| 22. 18 and 23 | 123 | 22. 18 and 23 | 56 |  |  |
